# How do you circumcise a nation? The Rwandan case study

**DOI:** 10.1186/s12916-014-0184-4

**Published:** 2014-10-06

**Authors:** Vincent Mutabazi, Jamie I Forrest, Nathan Ford, Edward J Mills

**Affiliations:** Ministry of Health, Republic of Rwanda, Rwanda Biomedical Centre-Medical Research Centre, PO Box 2717, Kigali, Rwanda; Global Evaluative Sciences, Calgary, Canada; Department of HIV/AIDS, World Health Organization, Geneva, Switzerland; Stanford Prevention Research Center, Stanford University, Stanford, USA

**Keywords:** Voluntary medical male circumcision, Rwanda, HIV/AIDS, Combination prevention, Scale-up

## Abstract

Voluntary medical male circumcision has been conclusively demonstrated to reduce the lifetime risk of male acquisition of HIV. The strategy has been adopted as a component of a comprehensive strategy towards achieving an AIDS-free generation. A number of countries in which prevalence of HIV is high and circumcision is low have been identified as a priority, where innovative approaches to scale-up are currently being explored. Rwanda, as one of the priority countries, has faced a number of challenges to successful scale-up. We discuss here how simplifications in the procedure, addressing a lack of healthcare infrastructure and mobilizing resources, and engaging communities of both men and women have permitted Rwanda to move forward with more optimism in its scale-up tactics. Examples from Rwanda are used to highlight how these barriers can and should be addressed.

## Background

For the first time since the beginning of the AIDS pandemic, the global scientific community has proposed a means to achieve an AIDS-free generation by implementing a multi-pronged, comprehensive strategy that targets both prevention and treatment of HIV [1]. One of the key pillars to achieving this goal includes scaling up voluntary medical male circumcision (VMMC) in sub-Saharan Africa. Multiple observational studies [2,3] and randomized trials (RCTs) [4-6] have conclusively demonstrated that VMMC can reduce the lifetime risk of male acquisition of HIV by about 60% [7]. The World Health Organization (WHO) and the Joint United Nations Programme on HIV/AIDS (UNAIDS) have endorsed innovative approaches to VMMC uptake in 13 priority countries in which HIV incidence remains high, but the prevalence of male circumcision is low [8]. Major implementing agencies and international donors have agreed to an action plan that aims to reach 80% coverage of VMMC in these 13 countries by 2015. Successful implementation of the plan will require performing about 20 million adult VMMC over the next 2 years. Reaching this target is estimated to avert 3.36 million new HIV infections in these priority countries, for a cost savings of up to US$16.5 billion [9]. However, implementation in most of these target countries has been slow, with prevalence of VMMC lagging far behind the goal of 80% coverage [10]. As of 2010, only 2.7% of the total number of VMMC procedures needed to reach this coverage level had been performed, and of all priority countries, only Kenya appears to be on track to achieving the 80% coverage [8].

Rwanda, as one of the identified priority countries, has set an ambitious goal of performing 700,000 additional VMMC procedures by 2015. Sufficiently convinced by the scientific evidence in support of VMMC, and with implementation support from the WHO, UNAIDS and the US President’s Emergency Plan for AIDS Relief (PEPFAR), the Rwandan government is promoting VMMC as a back-bone strategy of a comprehensive national HIV strategic plan [11]. However, Rwanda does not have a history of traditional circumcision, and until recently, the country has lagged behind other priority countries. By 2014, less than 10% of the target number of procedures had been performed. This is in comparison to neighboring Kenya, for example, which has achieved almost 50% VMMC coverage in some regions and up to 80% in others [12]. It may be that conflicting feasibility and optimization models for the scale-up of VMMC in Rwanda generated overly optimistic operational targets [13].

## Key challenges

As Rwanda and other priority countries embrace VMMC scale-up, three key challenges must be addressed to support the scale-up of circumcising millions of men. These include: 1) simplifying the VMMC procedure; 2) addressing a lack of health worker infrastructure and mobilizing resources in an over-burdened health system; and 3) engaging communities of men and women to communicate the benefits of the procedure. We discuss these challenges here in the context of Rwanda’s experiences of scaling up VMMC.

### Simplifying the VMMC procedure

VMMC scale-up began in many of the priority countries of sub-Saharan Africa in 2008 to 2009, with the forceps-guided surgical method being the predominant method of choice. There has been much discussion over the potential cost savings associated with using a device to increase the technical efficiency of the procedure [14]. However, there have been some concerns regarding the high rates of adverse events (AEs) associated with some of these devices, as well as the need for skilled operators, which may inhibit their successful scale-up [15,16].

This has led Rwanda to consider two newer devices, the PrePex (Circ MedTech Ltd, Israel) and the Shang Ring (Wuhu Santa Medical Devices Technology Co Ltd, China) devices [17]. The PrePex device is an FDA-approved and WHO pre-qualified non-surgical device [18]. It works by compressing the foreskin and cutting off blood circulation, after which the distal foreskin becomes necrotic, permitting easy and bloodless removal after 1 week. In the PrePex procedure, injected anesthetic or sutures are not necessary, and skilled health workers who are trained and certified can perform the procedure in a clean, non-sterile setting [19]. Procedure time with the PrePex device is five times faster than with the surgical technique, and the procedure is also safer [18]. In two cohort studies carried out in Zimbabwe and Rwanda using trained nurses, the AE rate was 0.6 to 0.9% in Zimbabwe [20] and 2.7%in Rwanda [18]. Expected side effects include clear exudates, localized mild edema in the wound area, and slough. The US Government, in partnership with the Bill and Melinda Gates Foundation, is now funding pilot evaluations in all priority African countries using the PrePex device [19].

The Shang Ring device, yet to receive pre-qualification by the WHO [21], involves injecting an anesthetic and cutting of the skin, although no suturing of the incision site is required. The device consists of an inner and outer ring; the outer ring applies constant pressure to the foreskin, thus replacing the need for sutures. The device must remain on the penis for 5 to 7 days. In RCTs in which the Shang Ring procedure was compared with conventional surgical procedure, AE rates related to the device were found to be 1% [21,22]. If the Shang Ring device receives final consideration in Rwanda, introducing variation in the procedure method would add options for clients, and may lead to increased demand.

### Addressing a lack of healthcare worker infrastructure and mobilizing resources

Like many countries in Africa, Rwanda struggles with a developing healthcare infrastructure, including a limited number of physicians. Simplification of the procedure by methods such as using devices described above, has allowed for VMMC to be task-shifted to lower-tier trained healthcare workers. Task-shifting of VMMC has been endorsed by the WHO as a component of scale-up strategies. Although several countries have not yet adopted this recommendation because of safety concerns, a meta-analysis of VMMC procedures performed by trained non-physician clinicians (for example nurses, midwives, surgical aides, and clinical officers) found a frequency of AEs similar to the rates reported when procedures were carried out by doctors or specialists [23].

One of the most critical tasks is building a national VMMC infrastructure with a focus on service providers. To overcome human resource constraints, the Rwandan government has sought to investigate task-shifting solutions for non-physician clinicians in clean, non-sterile settings, thus minimizing the burden on the healthcare system. The first stage of the Ministry of Health VMMC scale-up plan is to focus on 12 district hospitals, located in all four provinces and the city of Kigali. Each hospital has been instructed to send at least one team member (physician or nurse) to be trained at the device centre of excellence in Kigali. The teams will then return to their original sites to carry out PrePex VMMC under supervision. Once this trained team member gains sufficient experience, they will become a local trainer for available nurses at the district hospitals and surrounding health centers in that catchment area. District hospitals will organize the training in collaboration with the Ministry of Health.

The first phase of this new plan is underway, with four district hospitals trained and four more to be trained by fall of this year. When executing the first phase, it became clear the following factors may hinder achievement the national goal: 1) trained teams not fully dedicated to the PrePex VMMC program and 2) a lack of sufficient trainers and supervisors to allow rapid national expansion.

### Engaging communities of men and women to communicate the benefits of VMMC

Effective scale-up must include meaningful engagement of communities in the medical benefits of VMMC. There has been concern that VMMC would not be well received by men in many regions of Africa, given the low prevalence of traditionally practiced circumcision. However, the published evidence and observations from neighboring Uganda indicate that even in settings where there is no prior tradition, young men are willing to accept VMMC as a strategy to reduce HIV infection [24]. Strategies for demand creation need to be further developed. For example, programming that employs community mobilization, the use of mass media, and the inclusion of women have been shown to be effective in Kenya [12].

There remains debate over the impact of VMMC on behavioral disinhibition. VMMC must be accompanied by counseling and education about maintaining consistent condom use and other preventive methods. National leadership is necessary to help educate the population about the benefits and risks of circumcision. To overcome this challenge, Rwanda has now initiated feasibility and acceptability trials in children and newborn males. Infant and child male circumcision ensures that the wound is sufficiently healed before sexual debut, and thus reduces the risk of HIV transmission during healing period.

## Conclusions

Rwanda is facing a challenging target of 700,000 VMMC procedures by mid 2015. A major decision for the Ministry of Health for achieving this target while considering the limited resources of the country has been to use PrePex for all adult men seeking VMMC in the country. Surgical male circumcision will be only reserved to men who are not eligible for PrePex or for males under the age of 18 years.

This is a rare and historic opportunity to be a part of turning the tide on the pandemic of our generation, but to take programs to scale and to maximize the impact the intervention, an accelerated pace of service delivery is needed. For Rwanda to achieve its targets by 2015, it will require a bold strategy that focuses on the efficiency of task-shifting, judicious use of resources, and the leadership of civil society.
